# Expression of BCL-2 and Laminin in Rectosigmoid Hirschsprung Disease: Correlations with Hirschsprung−Associated Enterocolitis

**DOI:** 10.1038/s41390-025-03994-2

**Published:** 2025-04-14

**Authors:** Merve Dede, Fatih Celik, Ebrucan Bulut, Rumeysa Fatma Balaban, Nuseybe Huriyet, Ufuk Unal, Nesrin Ugras, Gulsah Cecener, Irfan Kiristioglu

**Affiliations:** 1 Bursa Uludag University Faculty of Medicine, Department of Pediatric Surgery, Bursa, Türkiye; 2 Bursa Uludag University Graduate School of Health Sciences, Department of Medical Biology, Bursa, Türkiye; 3 Bursa Uludag University Faculty of Medicine, Department of Pathology, Bursa, Türkiye; 4 Bursa Uludag University Faculty of Medicine, Department of Medical Biology, Bursa, Türkiye

## Abstract

**Background:**

Hirschsprung disease (HD) involves aganglionosis of the intestinal segment, with unclear etiology and challenging histopathological identification. The etiology of Hirschsprung−associated enterocolitis (HAEC) remains elusive. This study aims to explore the potential roles of Laminin and BCL-2 in the etiology of HD and HAEC by examining their expression levels.

**Methods:**

Tissues from 20 Rectosigmoid Hirschsprung patients (10 with and 10 without postoperative HAEC) and 10 controls were analyzed retrospectively. Protein expression was analyzed using immunohistochemistry, mRNA levels were measured using Real-Time PCR, and DNA mutations were found using Sanger Sequencing.

**Results:**

BCL-2 immunohistochemistry indicated decreased expression in HD patients’ aganglionic tissues compared to ganglionic tissues (*p* ≤ 0.001). *BCL-2* mRNA expression was significantly lower in HD patients’ tissues than in controls (*p* < 0.0001). Laminin immunohistochemistry revealed significant positive staining in ganglionic tissues, with most aganglionic tissues negative and some mildly positive (*p* < 0.016). There was no significant association between BCL-2, Laminin, and HAEC (*p* > 0.05). DNA sequencing discovered a novel *BCL-2* gene mutation in HD patients.

**Conclusion:**

BCL-2 and Laminin immunohistochemistry can differentiate ganglionic and aganglionic tissues. Reduced *BCL-2* mRNA expression in HD patients indicates a disease that affects the whole gut. More research is needed on the new *BCL-2* gene mutations.

**Impact statement:**

- This is the first study to examine the correlation between BCL-2 and Laminin expression changes and enterocolitis developing in HD. It is a previously unreported contribution to the literature that *BCL-2* mRNA expression was lower than expected in all intestinal tissues of HD patients, including the intestinal tissues considered to be healthy. Mutagenic changes have been detected in the *BCL-2* gene of some HD patients. A definitive novel mutation, which has not been reported in the literature before, was detected in one patient.

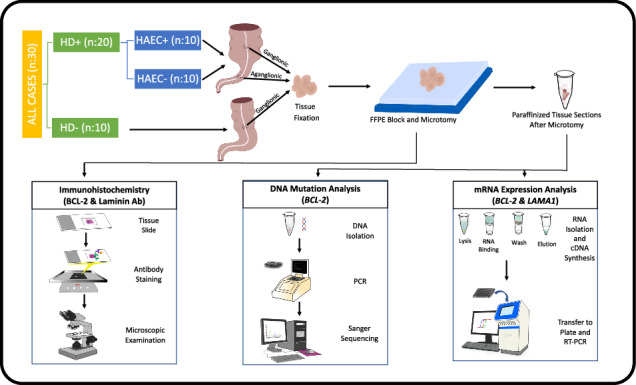

## Introduction

Hirschsprung disease (HD) is a congenital disease characterized by the absence of ganglion cells in the distal gastrointestinal tract, myenteric and submucosal nerve plexuses, the absence of normal peristaltic movements in the affected intestine, and functional intestinal obstruction.^1,2^ Hirschsprung−associated enterocolitis (HAEC) is the most serious and life-threatening complication of Hirschsprung disease. In addition to being seen in the pre-operative period, HAEC is a clinical condition that can be encountered in the postoperative period (after definitive pull-through surgery), and its etiology remains unclear.^3^

When embryological explanations regarding the etiopathogenesis of HD are examined, it is seen that neural differentiation and the enteric microenvironment play an important role in the progression of neural precursor cells to reach the intestinal wall in the craniocaudal direction.^4^

BCL-2 protein, one of the anti-apoptotic members of the BCL-2 family, an important protein family associated with apoptosis, is widely expressed in the developing central and peripheral nervous systems.^5^ There is increasing evidence that BCL-2 plays a critical role in the differentiation and development of neuronal cells.^6^ It appears that aganglionic tissues will have inadequate levels of BCL-2, a molecule with antiapoptotic features, which will cause problems in neuronal development.

It has been shown that extracellular matrix (ECM) molecules, one of the microenvironmental elements effective on neural migration, are biologically active and can change the fate of neural crest stem cells. Laminin is an important structure that plays a role in this regulation.^7^ The Laminin-1 isoform is well-recognized for promoting the development of enteric neurons.^8^ It has been discovered that the α1 subunit (*LAMA1*) of Laminin-1, expressed in intestinal tissue, supports neural development in this area.^9–11^ It was thought that the concentration of Laminin in the aganglionic intestines was significantly higher than in the ganglionic intestines, and the concentration in the ganglionic tissues was higher than in the intestines of the same age control group.^12^

While the current diagnosis and treatment of HD focuses on the aganglionic segment, the persistence of gastrointestinal motor dysfunction in some patients long after surgical correction indicates that morphological and functional abnormalities in the gut are not limited to the aganglionic segment.^10,13^

This study evaluates the correlation of changes in BCL-2 and Laminin expression levels in both aganglionic and ganglionic intestinal segments on the enteric nervous system and HAEC development in HD patients.

## Material And Methods

### Ethical approval

This thesis study, approved by the Uludag University Clinical Research Ethics Committee on June 8, 2022, and numbered 2022-12/7, was financed by the Scientific Research Projects (BAP) Unit of the Uludag University.

### Study design

A retrospective analysis was performed on the intestinal samples from 30 patients. Twenty of these children had operated in our institute with the histopathological diagnosis of Rectosigmoid HD. According to the Pastor Scale, ten of this HD group patients were with post-operative HAEC (HAEC+), and ten were not (HAEC−). On the other hand, 10 patients in the study’s control group had undergone surgery for an unrelated condition. The HD group’s aganglionic (G−) and ganglionic (G+) intestinal segments were examined. Just one intestinal sample was analyzed for the control group. Thus, 40 intestinal segments from 20 HD group patients and 10 intestinal segments from 10 control group patients were taken, and 50 intestinal samples were analyzed.

### Inclusion and exclusion criteria

The patients included in the HD group were required to meet the following criteria: undergoing surgery for rectosigmoid HD and receiving a histopathological diagnosis, being between the ages of 0 and 18 at the time of the surgery, to be followed up post-operatively, and having undergone surgery within the last 10 years.

To be included in the HAEC+ group, postoperative follow-up with at least 1 episode of enterocolitis was required. The patients of the HAEC group were randomly selected from those who had not had an attack of enterocolitis for at least 2 years after the surgery.

The control group patients, like the HD group, were between 0 and 18 years old at the time of surgery. Patients in the control group are required to have had their colons resected for a reason other than HD for them to participate and have undergone surgery during the last 10 years. Patients who underwent surgery due to inflammatory bowel diseases, anal atresia, intestinal atresia, or malignancy were not included in the control group. In the control group, 3 patients underwent colon resection due to trauma, 3 patients due to NEC, 2 patients due to dolichosigma, 1 patient due to volvulus, and 1 patient due to intussusception.

### Immunohistochemical staining and evaluation

The samples of all patients were subjected to immunohistological staining with appropriate protocols specific to BCL-2 and Laminin with a post-deparaffinization staining device *(Ventana Benchmark Ultra®, France)*. For BCL-2, Liquid Mouse Monoclonal Antibody BCL-2 *(Novocastra®, UK)* incubation was performed, and for Laminin, Laminin Ab-1 Antibody *(Thermoscientific®, Waltham, Massachusetts)* incubation was conducted. An expert pathologist examined intestinal tissue sections containing Auerbach and Meissner plexuses while evaluating the results of Laminin and BCL-2 immunohistochemical staining. Ganglion cells that exhibit cytoplasmic BCL-2 staining were considered to be positive. The absence of any staining was considered negative staining; mild staining was indicated by 1-2 ganglion cells and/or light yellow; severe staining was indicated by >3 ganglion cells and/or dark brown. Laminin immunohistochemistry assessed the basal membrane staining around the Auerbach and Meissner plexus. According to the staining intensity, it was accepted that 0 indicated no staining, 1+ indicated mild staining, and 2+ indicated severe staining.

### Molecular studies

#### DNA mutation

##### DNA isolation

DNA isolation from the deparaffinized tissue was carried out following the protocol of the tissue DNA isolation kit® (Hibrigen, Türkiye). The concentration of DNA materials was calculated, and a quality assessment was performed.

##### Polymerase Chain Reaction (PCR) analysis

Primers were designed according to *BCL-2* (ENST00000333681.5). Since Exon 1 does not code for a protein, Exon 2 and Exon 3 regions containing the coding regions were analyzed. The table indicates these exons’ characteristics **(**Table 1**)**. The relevant DNA segment was enzymatically amplified using the PCR. The suitability evaluation of PCR products of *BCL-2* gene exons was performed. After determining that the PCR products of the exons showed appropriate band characteristics (bright, single bands) in the agarose gel PCR image, the process was continued.

**Table 1:** Primers and Their Characteristics for the Exon 2 and 3 of the *BCL-2* Gene.

Exon number		Primer	Annealing Temperature (°C)	PCR product size (in base pairs)
Exon 2	F:	5’ TTGCTTTTCCTCTGGGAAG 3’	58	605
	R:	5’ GGGAAGCAACAACTCTGATT 3’		
Exon 3	F:	5’ TCATGGCCTCCAAAGAGCATT 3’	65	480
	R:	5’ TCGACGTTTTGCCTGAAGACT 3’		

##### Single-Strand Conformation Polymorphism (SSCP) analysis for BCL-2 gene

The resulting double-stranded DNA samples were provided with appropriate temperatures to separate the two strands and keep the strands separate.

##### Polyacrylamide gel electrophoresis

After all samples were loaded into each well, electrophoresis was performed at 1200 V (12 mA) for 8 h.

##### Silver staining and evaluation

The gel was deacidified and imaged. DNA sequence analysis was performed on samples with abnormal band appearance.

##### Mutation identification DNA sequence analysis

PCR products were purified using the *E.Z.N.A. Cycle Pure Kit® (Omega Bio-tek, Norcross, Georgia)*. Sequencing reactions were performed to remove chemicals and purify PCR products. After completing all necessary procedures, the samples were loaded into the sequencing device *(Beckman Coulter CEQ8000 Genetic Analysis System®, Brea, California)*. To analyze the detected changes, it was compared with the reference sequence of the *BCL-2* gene *(Ensemble, OMIM, Johns Hopkins University, Baltimore, Maryland)*.

#### mRNA expression

##### RNA isolation

*The E.Z.N.A. FFPE RNA Kit® (Omega Bio-tek, Norcross, Georgia)* protocol was followed for the isolation of total RNA from formalin-fixed, paraffin-embedded tissue sections.

##### cDNA (Complementary DNA) synthesis

*The High-Capacity cDNA Reverse Transcription Kit® (Thermo Fisher Scientific, Waltham, Massachusetts)* was used to synthesize cDNA from total RNA.

##### Gene expression analysis

*TaqMan Gene Expression Assays® (Thermo Fisher Scientific, Waltham, Massachusetts)* were used. An appropriate amount of RT-PCR mix and appropriate conditions were provided. RT-PCR analysis was performed using TaqMan probe primers on the *Step One Plus™ Real-Time PCR system® (Applied Biosystems, Foster City, California)*. The obtained data were evaluated using the “RT² Profiler PCR Array Data Analysis version 3.5.” program through the 2^−ΔΔCT^ method.

DNA mutation analysis was performed on all patients including the control group.

#### Statistical analyses

Descriptive statistical analyses (variance, median, etc.) of the obtained data and statistical evaluations based on quantitative results were conducted using the *SPSS Statistics Data Editor® v25 (IBM, Chicago, Illinois)* software package. All test results were accepted at a significance level of *p* < 0.05 with a 95% confidence interval. Graphical representation of statistical analyses was done using *GraphPad Prism 8® (Dotmatics, San Diego, California)* software. Normality test, t-test, and ANOVA (Post Hoc Test using Tukey) tests were used for the obtained quantitative data, and chi-square test was used for the categorical data.

A graphical abstract figure summarizing the materials and methods section is available as supplementary data.

## Results

The HD group exhibited a median age of 8 months with an interquartile range of 10.38 months. For the control group, the median age was 50 months with an interquartile range of 92.75 months. In the groups differentiated by the presence or absence of HAEC, the HAEC+ HD group had a median age of 12 months with an interquartile range of 7.75 months, and the HAEC− HD group had a median age of 2 months with an interquartile range of 10.63 months. In this study, the relationship between BCL-2 and laminin (*LAMA1*) expression levels and age was assessed using Spearman’s correlation analysis. The results indicate that there is no significant relationship between the expression levels of either marker and age. These findings suggest that the expressions studied do not exhibit significant changes dependent on age. In addition, it was found that sex had no statistically significant effect on the differences in expression levels.

### Results of the immunohistochemical study

**Fig. 1:**
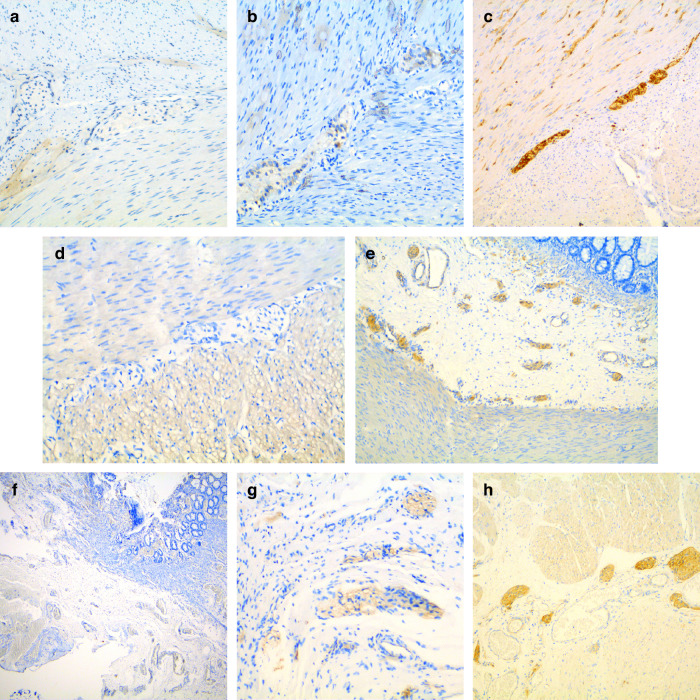
BCL-2 immunohistochemistry. **a** Negative staining in G− tissue (x200), **b** Mild staining in G+ tissue (x200), **c** Severe staining in G+ tissue (x200). Laminin immunohistochemistry (**d**) Negative staining in G+ tissue (x200), **e** Mild staining in G+ tissue (x200), **f** Negative staining in G− tissue (x100), **g** Mild staining in G− tissue (x200), **h** Severe staining in G− tissue (x200).

Numerical result data of BCL-2 and Laminin immunohistochemistry staining properties of HD and HAEC groups’ G+ and G− tissues and *p* values obtained in comparisons between groups are presented in tables (Table 2). To visualize the staining properties, the groups to which the tissues belong and their tissue properties are presented in figures (Fig. 1).

**Table 2:** Numerical result data of BCL-2 and Laminin immunohistochemistry staining status according to groups.

			BCL-2 Ab			Laminin Ab		
			Negative Staining	Positive Staining		Negative Staining	Positive Staining	
HD	HAEC	G	No Staining	Mild Staining	Severe Staining	No Staining	Mild Staining	Severe Staining
**+**	**+**	**+**	−	9	1	6	3	1
**+**	**+**	**-**	10	−	−	2	5	3
**+**	−	**+**	−	6	4	8	2	−
**+**	−	**-**	9	1	−	3	6	1
**-**			−	9	1	−	5	5
**+**		**+**	−	15	5	14	5	1
**+**		−	19	1	−	5	11	4

*p* values in comparison between groups:

BCL-2:

HD + , HAEC + , G+ & HD + , HAEC−, G+ → *p* = 0.153

HD + , HAEC + , G+ & HD− → *p* = 1

HD + , HAEC−, G+ & HD− → *p* = 0.121

HD + , G+ & HD + , G− → *p* < 0.001

Laminin:

HD + , HAEC + , G+ & HD + , HAEC−, G+ → p:0.541

HD + , HAEC + , G+ & HD− → *p* = 0.451

HD + , HAEC−, G+ & HD− → *p* = 0.160

HD + , G+ & HD + , G− → *p* < 0.016

### Results of the genetic study

mRNA Expression Levels: *BCL-2* mRNA expression levels were significantly lower in all HAEC+ and HAEC− HD children, not only in G− intestinal tissues but also in G+ intestinal tissues, compared to the control group (*p* < 0.0001). Although there was no statistically significant difference, it was determined that the average *BCL-2* expression levels in the G+ intestinal tissues of the HAEC+ group were slightly lower than in the G+ tissues of the HAEC− group.

No significant difference was observed between groups regarding *LAMA1* mRNA expression levels in the analyses. Although no statistically significant difference could be determined, the average *LAMA1* expression levels in the G+ tissues of the HAEC+ group were determined to be slightly lower than in the G+ tissues of the HAEC− group.

**Fig. 2:**
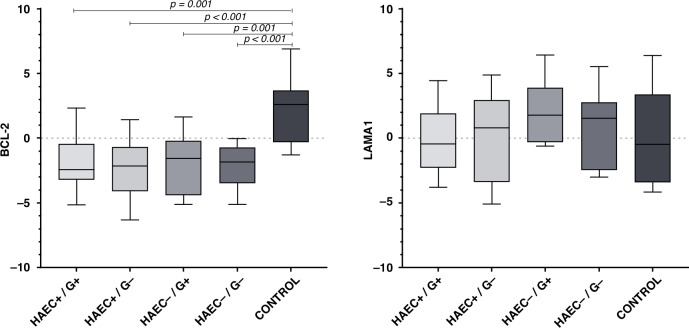
Box plot expressing the distribution of expression values of the different tissues at all groups (maximum, minimum, average values, etc.). *BCL-2* mRNA expression levels were significantly lower in all intestinal tissues examined in all HD patients, both G+ and G−, compared to the control group (*p* < 0.001). No statistically significant difference was observed between the groups in terms of *LAMA1* mRNA expression levels.

**Fig. 3:**
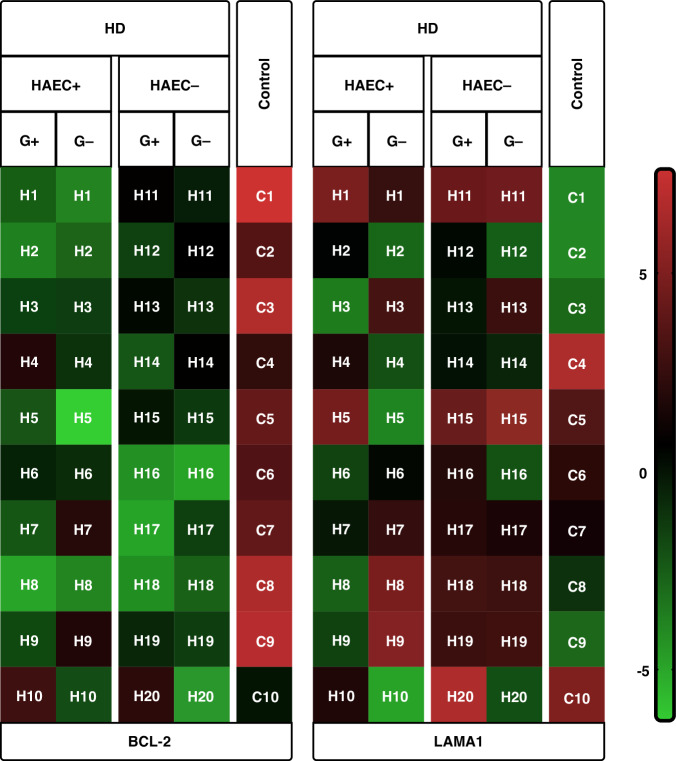
This is a heat map graph showing the mRNA expression differences of all tissues examined in all groups on a patient basis. Those colored close to red indicate an increase in expression, and those colored close to green indicate a decrease in expression. The black color at the limit value suggests that there is no change.

The graphs provide information about the *BCL-2* and *LAMA1* mRNA expression levels of tissues examined according to groups (Figs. 2, 3).

Expression levels between G+ and G− tissues of HD groups did not show a statistically significant change. However, a statistically significant similarity was observed between BCL-2 immunohistochemistry and *BCL-2* mRNA expression (*p* = 0.019). The same was not true for Laminin and *LAMA1* (*p* = 0.563).

#### DNA Mutation Analyses

**Fig. 4:**
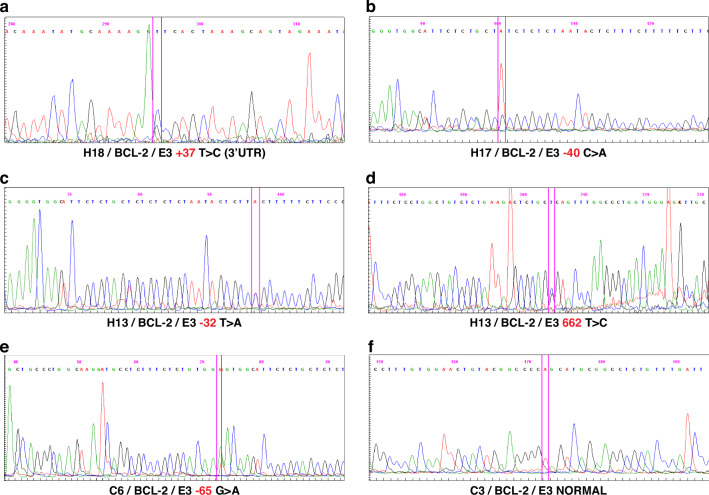
Sequence analysis results of the 3rd exon of the *BCL-2* gene are presented below the images of the patients, indicating the patient number (H), the gene under mutation analysis (*BCL-2*), the exon under investigation (E), the base sequence of the current change, and the base change. **a** Thymine (T) > Cytosine (C) change was detected at +37 in the 3’UTR part of Exon 3 in the *BCL-2* gene of patient number 18. **b** Cytosine (C) >Adenine (A) change was detected in the -40 (intronic) part of Exon 3 of the *BCL-2* gene of patient number 17. **c**, **d** Thymine (T) > Cytosine (C) change in the 662nd base of the 3rd exon of the *BCL-2* gene of patient number 13 and Thymine (T) >Adenine (A) change in the −32 (intronic) part were detected. **e** Guanine (G) >Adenine (A) change was detected in the -65 (intronic) part of Exon 3 of the *BCL-2* gene of patient number 6 in the control group. **f** Normal sequence images of a control group patient (C3) are given in figure.

After determining the suitability of PCR products in agarose gel electrophoresis, SSCP analysis was applied to the PCR products of all samples. With SSCP analysis, imaging and evaluation of *BCL-2* Exon 2 and Exon 3 of all patients included were performed. As a result of SSCP, it was decided to conduct DNA sequence analysis due to the different band features detected in 2 HD patients for Exon 3, 4 HD patients for Exon 2, and 2 control group patients. DNA sequence analysis was performed on patients who showed different bands in SSCP and suspected mutation. The detected mutagenic nucleotide changes are shown in figure (Fig. 4).

The thymine to cytosine (T > C) change at base 662 of the 3rd exon of patient number 13’s *BCL-2* gene is a novel change that has not been described in the literature. This change causes the transformation of “leucine” into “proline” in amino acid expression with the CTC > CCC codon change. *BCL-2* mRNA expression in this patient’s G− tissue was reduced 6-fold compared to G+ tissue [log2(2 − ^ΔΔCt^ *BCL-2*) value = −0.25 (in G+ tissue) / −1.52 (in G− tissue)].

Tables for Spearman correlation analysis evaluating the relationship between BCL-2 and laminin (*LAMA1*) expression levels and age, tables illustrating the age distribution characteristics by group (in months), tables presenting statistical data on the relationships between age and the staining classifications used in protein expression evaluation (negative, mild, severe), tables showing age distribution characteristics of patients when grouped according to staining properties, and tables demonstrating the relationships between negative and positive staining classifications used in protein expression evaluation with age and mRNA expression levels, along with a statistical table of within-group comparisons for mRNA expression levels, are available as supplementary data.

## Discussion

The main problem in HD is that intrauterine neuronal migration is interrupted for any reason, and the distal intestines remain aganglionic.^1,2^ Laminin and BCL-2 play a role in this mechanism.^5,6,14–16^ The study conducted by Ge et al. in 2017 with tissue samples taken from narrow, transitional, and dilated segments of 15 HD and 5 control patients confirmed that the highest *BCL-2* expression levels were in the G + segment and were similar to the control group. It was found to be low in G− tissues, consistent with the literature.^17^ In our study, although immunohistochemically determined that BCL-2 expression in the G− tissues of patients in the HD group was significantly reduced compared to G+ tissues, the significance of this decrease could not be determined statistically at the mRNA level by RT-PCR. In our study, we made a significant contribution to the literature in elucidating the molecular pathophysiology of the disease by discovering that *BCL-2* mRNA expression was reduced in both G− and G+ tissues in HD patients compared to the control group.

The decreased expression of BCL-2 in the G+ segments of HD patients, which are generally considered healthy, suggests that the pathology affecting BCL-2 expression is not limited to the diseased intestinal region, but rather encompasses the entire intestines and other organs of these patients.

In the study by Li et al. that evaluated Laminin and RET gene expression, it was found that Laminin was expressed in all segments, with intense expression in the G− segment, gradually decreasing towards the G+ segment. They stated that the quantitative *LAMA1* expression level determined by RT-PCR was twofold higher in the G− segment compared to the G+ segment. Based on these findings, they suggested that highly increased Laminin expression in the G−segment may cause early differentiation, early maturation, and early termination of migration of enteric nerve cells.^18^ In another experimental study, in mice with Hirschsprung, although there was colonization up to the distal colon, aganglionosis was observed from the proximal colon, and *LAMA1* levels in the G−proximal and distal colons were significantly increased compared to the control group.^11^ Similarly, in our study, Laminin protein was increased immunohistochemically in G− tissues compared to G+ tissues (*p* < 0.016). Although *LAMA1* mRNA expression levels tend to be high in G− tissue and low in G+ tissue, similar to this study, no statistical significance was detected. Researchers who participated in our research and are experts in molecular biology predict that intertissue expression differences for both BCL-2 and Laminin, which were detected at the protein level by immunohistochemistry but not at the mRNA level by molecular methods, result from the reflection of mRNA into protein synthesis due to post-transcriptional or post-translational regulations. Wester et al. stated that anti-BCL-2 is suitable for imaging the nerve cell body in diagnosing HD, mainly in G+ tissues. They also suggested that BCL-2 supports the survival of enteric neurons throughout life, starting from the fetal period.^5^ Our study, focused exclusively on pediatric patient series, supports the observed findings. However, despite the broad age range of 0–18 years specified in our inclusion criteria, our analysis revealed that age and sex did not statistically significantly impact expression levels among the groups. The absence of a significant age effect could be attributed to the similar age distributions within both the HD groups and the control group. Due to the non-normal distribution of age within these groups, non-parametric tests were applied to ensure appropriate statistical analyses. Where data showed normal distribution, the t-test was used, whereas the Mann-Whitney U test was utilized for data that did not show normal distribution. Additionally, the limited number of patients in our study could have impacted the sensitivity of our statistical analyses. Therefore, to accurately assess the potential effects of age and sex on expression levels, the necessary non-parametric tests were rigorously applied. Izadi B et al. published a study in which they identified ganglion cells with BCL-2 immunohistochemistry and compared them with H&E staining. They reported that BCL-2 had higher sensitivity, specificity, and predictive values for the diagnosis of HD.^19^ In our study, the diagnosis was easily confirmed with BCL-2 immunohistochemistry in patients diagnosed with H&E histochemistry.

The fact that HAEC can be seen both in the pre-operative and post-operative periods suggests that there may be reasons other than the presence or absence of ganglion cells in its etiology.^1,2^ Although studies examining BCL-2 in the etiology of HD are included in the literature, it has been determined that there are no studies examining the relationship of BCL-2 with the development of HAEC.^5,6,15,16^ Our study is important and unique because it is the first to investigate this issue. In this study, G+ and G− tissues of patients with HAEC were compared among themselves using immunohistochemical and molecular methods. Likewise, this comparison was made in groups that did not have HAEC. No statistically significant difference was detected between the HAEC+ and HAEC− groups. It aimed to investigate the possible effects of BCL-2 and Laminin expression levels differences on the etiology of HAEC developing in the postoperative period by comparing the G+ tissues left in the patient after surgery in the HAEC+ and HAEC− groups. In this analysis, G− tissues were disabled. It was found that *BCL-2* mRNA expression levels in G+ intestinal segments tended to be lower in the HAEC group than in the non-HAEC group. The mean *LAMA1* mRNA expression levels in G+ tissues of the HAEC group were lower than in the non-HAEC group. However, the results of both comparisons were not statistically significant. The lack of significance may be due to insufficient sample size. This study is valuable because it sheds light on the etiology of HD and is a preliminary study that will contribute to the understanding of HAEC at the molecular level.

Mutation analysis of the *BCL-2* gene was performed in all patients. As a result of the molecular genetic study, DNA sequence analysis of the *BCL-2* gene revealed an apparent mutagenic change in 3 of 20 HD patients and a possible mutagenic change in 2. The mutagenic change detected in the *BCL-2* gene in one of the three patients with an apparent mutagenic change is a new (novel) change that has not been described in the literature. One of the cases in which mutagenic change was detected was in the patient group with HAEC; the other four patients were in the patient group without HAEC. None of these cases in which mutations were detected have known syndromic features.

## Conclusions

BCL-2 and Laminin immunohistochemistry can be used to diagnose Hirschsprung Disease. The fact that *BCL-2* mRNA expression is lower than expected in all intestinal tissues of Hirschsprung patients suggests that the *BCL-2* mRNA expression problem may affect the entire intestine and even other organs. Our study found no statistical relationship between BCL-2 and Laminin expression differences and HAEC development. Mutagenic changes have been detected in the *BCL-2* gene of some HD patients. Further analysis is needed to determine their impact on the clinical picture. The results obtained have the potential to be meaningful in studies with more extensive patient series.

## Supplementary information

Age–Related Data

Age and Staining Patterns in Protein Expression Analysis

Age mRNA Protein Statistics

Statistical Table of Inter-Group Comparisons for mRNA Expression Levels

## Data Availability

The datasets used and/or analyses during the current study are available from the corresponding author on reasonable request.
