# Supplementary material for: Expression of BCL-2 and Laminin in Rectosigmoid Hirschsprung Disease: Correlations with Hirschsprung−Associated Enterocolitis
Source: Pediatr Res. 2025 Apr 14;99(2):759–66. doi: 10.1038/s41390-025-03994-2 (PMC12956551; doi:10.1038/s41390-025-03994-2)
Supplement: Supplementary file 1 — Age–Related Data [file 41390_2025_3994_MOESM1_ESM.pdf]

Age and Staining Patterns in Protein Expression Analysis

The tables below present statistical data on the relationships between age and the negative-mild-severe staining classifications used in protein expression evaluation. Additionally, these tables show the age distribution characteristics of the patients when grouped according to staining properties.

Post Hoc Tests

Multiple Comparisons

Dependent Variable: Age

	(I) BCL-2	(J) BCL-2	Mean Difference (I-J)	Std. Error	Sig.
Tukey HSD	Negative staining	Mild staining	,92105	3,24063	,956
		Severe staining	,62105	4,80038	,991
	Mild staining	Negative staining	-,92105	3,24063	,956
		Severe staining	-,30000	4,89324	,998
	Severe staining	Negative staining	-,62105	4,80038	,991
		Mild staining	,30000	4,89324	,998

Multiple Comparisons

Dependent Variable: Age

	(I) BCL-2	(J) BCL-2	95% Confidence Interval	
			Lower Bound	Upper Bound
Tukey HSD	Negative staining	Mild staining	-6,9909	8,8330
		Severe staining	-11,0990	12,3411
	Mild staining	Negative staining	-8,8330	6,9909
		Severe staining	-12,2468	11,6468
	Severe staining	Negative staining	-12,3411	11,0990
		Mild staining	-11,6468	12,2468

Post Hoc Tests

Multiple Comparisons

Dependent Variable: Age

	(I) Laminin	(J) Laminin	Mean Difference (I-J)	Std. Error	Sig.
Tukey HSD	Negative staining	Mild staining	-7,60362*	2,99334	,040
		Severe staining	-3,04737	4,43406	,772
	Mild staining	Negative staining	7,60362*	2,99334	,040
		Severe staining	4,55625	4,51984	,577
	Severe staining	Negative staining	3,04737	4,43406	,772
		Mild staining	-4,55625	4,51984	,577

Multiple Comparisons

Dependent Variable: Age

	(I) Laminin	(J) Laminin	95% Confidence Interval	
			Lower Bound	Upper Bound
Tukey HSD	Negative staining	Mild staining	-14,9118	-,2954
		Severe staining	-13,8731	7,7783
	Mild staining	Negative staining	,2954	14,9118
		Severe staining	-6,4789	15,5914
	Severe staining	Negative staining	-7,7783	13,8731
		Mild staining	-15,5914	6,4789

*. The mean difference is significant at the 0.05 level.

Descriptives

	BCL-2			Statistic	Std. Error
Age (month)	Negative staining	Mean		9,9211	2,17349
		95% Confidence Interval for Mean	Lower Bound	5,3547	
			Upper Bound	14,4874	
		5% Trimmed Mean		8,9678	
		Median		8,0000	
		Variance		89,757	
		Std. Deviation		9,47403	
		Minimum		1,00	
		Maximum		36,00	
		Range		35,00	
		Interquartile Range		10,00	
		Skewness		1,404	,524
		Kurtosis		1,971	1,014
	Mild staining	Mean		9,0000	2,41911
		95% Confidence Interval for Mean	Lower Bound	3,8438	
			Upper Bound	14,1562	
		5% Trimmed Mean		7,9444	
		Median		6,5000	
		Variance		93,633	
		Std. Deviation		9,67643	
		Minimum		1,00	
		Maximum		36,00	
		Range		35,00	
		Interquartile Range		10,38	
		Skewness		1,699	,564
		Kurtosis		3,164	1,091
	Severe staining	Mean		9,3000	4,21189
		95% Confidence Interval for Mean	Lower Bound	-2,3941	
			Upper Bound	20,9941	
		5% Trimmed Mean		8,9444	
		Median		8,0000	
		Variance		88,700	
		Std. Deviation		9,41807	
		Minimum		1,00	
		Maximum		24,00	
		Range		23,00	
		Interquartile Range		16,75	
		Skewness		1,071	,913
		Kurtosis		,770	2,000

Descriptives

	Laminin			Statistic	Std. Error
Age (month)	Negative staining	Mean		6,0526	1,42549
		95% Confidence Interval for Mean	Lower Bound	3,0578	
			Upper Bound	9,0475	
		5% Trimmed Mean		5,3363	
		Median		2,5000	
		Variance		38,608	
		Std. Deviation		6,21355	
		Minimum		1,00	
		Maximum		24,00	
		Range		23,00	
		Interquartile Range		11,00	
		Skewness		1,450	,524
		Kurtosis		2,351	1,014
	Mild staining	Mean		13,6563	2,95829
		95% Confidence Interval for Mean	Lower Bound	7,3508	
			Upper Bound	19,9617	
		5% Trimmed Mean		13,1181	
		Median		12,0000	
		Variance		140,024	
		Std. Deviation		11,83317	
		Minimum		1,00	
		Maximum		36,00	
		Range		35,00	
		Interquartile Range		21,88	
		Skewness		,772	,564
		Kurtosis		-,482	1,091
	Severe staining	Mean		9,1000	2,05183
		95% Confidence Interval for Mean	Lower Bound	3,4032	
			Upper Bound	14,7968	
		5% Trimmed Mean		9,3611	
		Median		12,0000	
		Variance		21,050	
		Std. Deviation		4,58803	
		Minimum		1,50	
		Maximum		12,00	
		Range		10,50	
		Interquartile Range		7,25	
		Skewness		-1,584	,913
		Kurtosis		2,014	2,000
