# Supplementary material for: Expression of BCL-2 and Laminin in Rectosigmoid Hirschsprung Disease: Correlations with Hirschsprung−Associated Enterocolitis
Source: Pediatr Res. 2025 Apr 14;99(2):759–66. doi: 10.1038/s41390-025-03994-2 (PMC12956551; doi:10.1038/s41390-025-03994-2)
Supplement: Supplementary file 2 — Age and Staining Patterns in Protein Expression Analysis [file 41390_2025_3994_MOESM2_ESM.pdf]

Statistical Data on Age - mRNA Expression Analysis - Protein Staining

In the tables presented below, instead of using the negative-mild-severe staining classification for protein expression evaluation, data are shown for the relationships of negative and positive staining classifications with age and mRNA expression levels.

When analyzing the data, for those conforming to a normal distribution, the independent t-test was utilized, whereas the Mann-Whitney U Test was applied for non-normally distributed data.

Table 1-2: In the first table, categories for negative and positive staining have been established. In the second table, the p-value for the comparison of negative and positive BCL-2 staining by age is indicated as 0.128.

Group Statistics

	BCL-2	N	Mean	Std. Deviation	Std. Error Mean
Age	Negative staining	19	9,9211	9,47403	2,17349
	Positive staining	31	25,3710	42,70967	7,67088

		t-test for Equality of Means			
		Sig. (2-tailed)	Mean Difference	Std. Error Difference	95% Confidence Interval of the Difference
					Lower
Age	Equal variances assumed	,128	-15,44992	9,98187	-35,51980
	Equal variances not assumed	,061	-15,44992	7,97286	-31,64173

Table 3-4: In the first table, categories for negative and positive staining have been established. In the second table, the p-value for the comparison of negative and positive Laminin staining by age is indicated as 0.045.

Group Statistics

	Laminin	N	Mean	Std. Deviation	Std. Error Mean
Age	Negative staining	24	9,2917	14,07582	2,87321
	Positive staining	26	28,9231	44,62481	8,75165

Independent Samples Test

		t-test for Equality of Means			
					95% Confidence Interval of the Difference
		Sig. (2-tailed)	Mean Difference	Std. Error Difference	Lower
Age	Equal variances assumed	,045	-19,63141	9,52439	-38,78148
	Equal variances not assumed	,041	-19,63141	9,21122	-38,43551

Table 5-6: In the first table, categories for negative and positive staining have been established. In the second table, the p-value for the comparison of negative and positive Laminin staining based on *LAMA1* mRNA expression levels is indicated as 0.871

Group Statistics

	Laminin	N	Mean	Std. Deviation	Std. Error Mean
LAMA1_ex	Negative staining	24	,6460	2,94164	,60046
	Positive staining	26	,4987	3,37913	,66270

Independent Samples Test

		t-test for Equality of Means		
		Sig. (2-tailed)	Mean Difference	Std. Error Difference
LAMA1_ex	Equal variances assumed	,871	,14731	,89932
	Equal variances not assumed	,870	,14731	,89427

Table 7-8: In the first table, categories for negative and positive staining have been established. In the second table, the p-value for the comparison of negative and positive BCL-2 staining based on *BCL-2* mRNA expression levels is indicated as 0.019

Group Statistics

	BCL-2	N	Mean	Std. Deviation	Std. Error Mean
BCL-2_ex	Negative staining	19	-2,1895	2,09863	,48146
	Positive staining	31	,0652	3,67641	,66030

Independent Samples Test

		t-test for Equality of Means		
		Sig. (2-tailed)	Mean Difference	Std. Error Difference
BCL-2_ex	Equal variances assumed	,019	-2,25463	,92591
	Equal variances not assumed	,008	-2,25463	,81719
