# Supplementary material for: Expression of BCL-2 and Laminin in Rectosigmoid Hirschsprung Disease: Correlations with Hirschsprung−Associated Enterocolitis
Source: Pediatr Res. 2025 Apr 14;99(2):759–66. doi: 10.1038/s41390-025-03994-2 (PMC12956551; doi:10.1038/s41390-025-03994-2)
Supplement: Supplementary file 3 — Age mRNA Protein Statistics [file 41390_2025_3994_MOESM3_ESM.pdf]

Age – Related Data

Tables displaying the distribution characteristics of age (in months) by group can be accessed below. **Table 1** presents data for the HD and control groups. **Table 2** additionally provides age-related data for groups with and without HAEC.

Table1:

Descriptives

				Statistic	Std. Error
Age (month)	HD Group	Mean		9,4750	1,47250
		95% Confidence Interval for Mean	Lower Bound	6,4966	
			Upper Bound	12,4534	
		5% Trimmed Mean		8,4722	
		Median		8,0000	
		Variance		86,730	
		Std. Deviation		9,31290	
		Minimum		1,00	
		Maximum		36,00	
		Range		35,00	
		Interquartile Range		10,38	
	Control Group	Mean		59,6000	19,90768
		95% Confidence Interval for Mean	Lower Bound	14,5657	
			Upper Bound	104,6343	
		5% Trimmed Mean		56,1667	
		Median		50,0000	
		Variance		3963,156	
		Std. Deviation		62,95360	
		Minimum		1,00	
		Maximum		180,00	
		Range		179,00	
		Interquartile Range		92,75	

Table 2:

Descriptives

	Group			Statistic	Std. Error
Age (month)	HD+ / HAEC+	Mean		13,0000	3,18329
		95% Confidence Interval for Mean	Lower Bound	5,7989	
			Upper Bound	20,2011	
		5% Trimmed Mean		12,3889	
		Median		12,0000	
		Variance		101,333	
		Std. Deviation		10,06645	
		Minimum		1,00	
		Maximum		36,00	
		Range		35,00	
		Interquartile Range		7,75	
		Skewness		1,498	,687
		Kurtosis		2,474	1,334
	HD+ / HAEC-	Mean		5,9500	2,42836
		95% Confidence Interval for Mean	Lower Bound	,4567	
			Upper Bound	11,4433	
		5% Trimmed Mean		5,2222	
		Median		2,0000	
		Variance		58,969	
		Std. Deviation		7,67916	
		Minimum		1,00	
		Maximum		24,00	
		Range		23,00	
		Interquartile Range		10,63	
		Skewness		1,761	,687
		Kurtosis		2,655	1,334
	HD-	Mean		59,6000	19,90768
		95% Confidence Interval for Mean	Lower Bound	14,5657	
			Upper Bound	104,6343	
		5% Trimmed Mean		56,1667	
		Median		50,0000	
		Variance		3963,156	
		Std. Deviation		62,95360	
		Minimum		1,00	
		Maximum		180,00	
		Range		179,00	
		Interquartile Range		92,75	
		Skewness		,906	,687
		Kurtosis		-,132	1,334
