# Supplementary material for: Expression of BCL-2 and Laminin in Rectosigmoid Hirschsprung Disease: Correlations with Hirschsprung−Associated Enterocolitis
Source: Pediatr Res. 2025 Apr 14;99(2):759–66. doi: 10.1038/s41390-025-03994-2 (PMC12956551; doi:10.1038/s41390-025-03994-2)
Supplement: Supplementary file 4 — Statistical Table of Inter-Group Comparisons for mRNA Expression Levels [file 41390_2025_3994_MOESM4_ESM.pdf]

One Way Anova					
**Tukey's multiple comparisons test**	**Mean Diff.**	**95,00% CI of diff.**	**Significant?**	**Summary**	**Adjusted P Value**
BCL-2(HD+ / HAEC+ / G+) vs. BCL-2(HD+ / HAEC+ / G-)	0,316	-3,681 to 4,313	No	ns	>0,9999
BCL-2(HD+ / HAEC+ / G+) vs. BCL-2(HD+ / HAEC- / G+)	-0,002	-3,999 to 3,995	No	ns	>0,9999
BCL-2(HD+ / HAEC+ / G+) vs. BCL-2(HD+ / HAEC- / G-)	0,216	-3,781 to 4,213	No	ns	>0,9999
BCL-2(HD+ / HAEC+ / G+) vs. BCL-2(HD-)	-6,122	-10,12 to -2,125	Yes	***	0,0001
BCL-2(HD+ / HAEC+ / G+) vs. LAMA1(HD+ / HAEC+ / G+)	-1,826	-5,823 to 2,171	No	ns	0,8957
BCL-2(HD+ / HAEC+ / G+) vs. LAMA1(HD+ / HAEC+ / G-)	-1,963	-5,960 to 2,034	No	ns	0,8479
BCL-2(HD+ / HAEC+ / G+) vs. LAMA1(HD+ / HAEC- / G-)	-3,958	-7,955 to 0,03896	No	ns	0,0545
BCL-2(HD+ / HAEC+ / G+) vs. LAMA1(HD+ / HAEC- / G-)	-2,74	-6,737 to 1,257	No	ns	0,4484
BCL-2(HD+ / HAEC+ / G+) vs. LAMA1(HD-)	-1,91	-5,907 to 2,087	No	ns	0,8676
BCL-2(HD+ / HAEC+ / G-) vs. BCL-2(HD+ / HAEC- / G+)	-0,318	-4,315 to 3,679	No	ns	>0,9999
BCL-2(HD+ / HAEC+ / G-) vs. BCL-2(HD+ / HAEC- / G-)	-0,1	-4,097 to 3,897	No	ns	>0,9999
BCL-2(HD+ / HAEC+ / G-) vs. BCL-2(HD-)	-6,438	-10,43 to -2,441	Yes	****	<0,0001
BCL-2(HD+ / HAEC+ / G-) vs. LAMA1(HD+ / HAEC+ / G+)	-2,142	-6,139 to 1,855	No	ns	0,7704
BCL-2(HD+ / HAEC+ / G-) vs. LAMA1(HD+ / HAEC+ / G-)	-2,279	-6,276 to 1,718	No	ns	0,7018
BCL-2(HD+ / HAEC+ / G-) vs. LAMA1(HD+ / HAEC- / G-)	-4,274	-8,271 to -0,2770	Yes	*	0,0263
BCL-2(HD+ / HAEC+ / G-) vs. LAMA1(HD+ / HAEC- / G-)	-3,056	-7,053 to 0,9410	No	ns	0,2931
BCL-2(HD+ / HAEC+ / G-) vs. LAMA1(HD-)	-2,226	-6,223 to 1,771	No	ns	0,7291
BCL-2(HD+ / HAEC- / G+) vs. BCL-2(HD+ / HAEC- / G-)	0,218	-3,779 to 4,215	No	ns	>0,9999
BCL-2(HD+ / HAEC- / G+) vs. BCL-2(HD-)	-6,12	-10,12 to -2,123	Yes	***	0,0001
BCL-2(HD+ / HAEC- / G+) vs. LAMA1(HD+ / HAEC+ / G+)	-1,824	-5,821 to 2,173	No	ns	0,8963
BCL-2(HD+ / HAEC- / G+) vs. LAMA1(HD+ / HAEC+ / G-)	-1,961	-5,958 to 2,036	No	ns	0,8487
BCL-2(HD+ / HAEC- / G+) vs. LAMA1(HD+ / HAEC- / G-)	-3,956	-7,953 to 0,04096	No	ns	0,0547
BCL-2(HD+ / HAEC- / G+) vs. LAMA1(HD+ / HAEC- / G-)	-2,738	-6,735 to 1,259	No	ns	0,4495
BCL-2(HD+ / HAEC- / G+) vs. LAMA1(HD-)	-1,908	-5,905 to 2,089	No	ns	0,8683
BCL-2(HD+ / HAEC- / G-) vs. BCL-2(HD-)	-6,338	-10,33 to -2,341	Yes	****	<0,0001
BCL-2(HD+ / HAEC- / G-) vs. LAMA1(HD+ / HAEC+ / G+)	-2,042	-6,039 to 1,955	No	ns	0,8156
BCL-2(HD+ / HAEC- / G-) vs. LAMA1(HD+ / HAEC+ / G-)	-2,179	-6,176 to 1,818	No	ns	0,7526
BCL-2(HD+ / HAEC- / G-) vs. LAMA1(HD+ / HAEC- / G-)	-4,174	-8,171 to -0,1770	Yes	*	0,0334
BCL-2(HD+ / HAEC- / G-) vs. LAMA1(HD+ / HAEC- / G-)	-2,956	-6,953 to 1,041	No	ns	0,3387
BCL-2(HD+ / HAEC- / G-) vs. LAMA1(HD-)	-2,126	-6,123 to 1,871	No	ns	0,778
BCL-2(HD-) vs. LAMA1(HD+ / HAEC+ / G+)	4,296	0,2990 to 8,293	Yes	*	0,0249
BCL-2(HD-) vs. LAMA1(HD+ / HAEC+ / G-)	4,159	0,1620 to 8,156	Yes	*	0,0345
BCL-2(HD-) vs. LAMA1(HD+ / HAEC- / G-)	2,164	-1,833 to 6,161	No	ns	0,7599
BCL-2(HD-) vs. LAMA1(HD+ / HAEC- / G-)	3,382	-0,6150 to 7,379	No	ns	0,172
BCL-2(HD-) vs. LAMA1(HD-)	4,212	0,2150 to 8,209	Yes	*	0,0305
LAMA1(HD+ / HAEC+ / G+) vs. LAMA1(HD+ / HAEC+ / G-)	-0,137	-4,134 to 3,860	No	ns	>0,9999
LAMA1(HD+ / HAEC+ / G+) vs. LAMA1(HD+ / HAEC- / G-)	-2,132	-6,129 to 1,865	No	ns	0,7751
LAMA1(HD+ / HAEC+ / G+) vs. LAMA1(HD+ / HAEC- / G-)	-0,914	-4,911 to 3,083	No	ns	0,9991
LAMA1(HD+ / HAEC+ / G+) vs. LAMA1(HD-)	-0,084	-4,081 to 3,913	No	ns	>0,9999
LAMA1(HD+ / HAEC+ / G-) vs. LAMA1(HD+ / HAEC- / G-)	-1,995	-5,992 to 2,002	No	ns	0,8352
LAMA1(HD+ / HAEC+ / G-) vs. LAMA1(HD+ / HAEC- / G-)	-0,777	-4,774 to 3,220	No	ns	0,9998
LAMA1(HD+ / HAEC+ / G-) vs. LAMA1(HD-)	0,053	-3,944 to 4,050	No	ns	>0,9999
LAMA1(HD+ / HAEC- / G-) vs. LAMA1(HD+ / HAEC- / G-)	1,218	-2,779 to 5,215	No	ns	0,9922
LAMA1(HD+ / HAEC- / G-) vs. LAMA1(HD-)	2,048	-1,949 to 6,045	No	ns	0,8131
LAMA1(HD+ / HAEC- / G-) vs. LAMA1(HD-)	0,83	-3,167 to 4,827	No	ns	0,9996
